# Antibacterial Activities and Synergistic Interaction of Citrus Essential Oils and Limonene with Gentamicin against Clinically Isolated Methicillin-Resistant *Staphylococcus aureus*

**DOI:** 10.1155/2022/8418287

**Published:** 2022-02-28

**Authors:** Apichai Sreepian, Supaluk Popruk, Daranee Nutalai, Chayanit Phutthanu, Preeyaporn M. Sreepian

**Affiliations:** ^1^Faculty of Medical Technology, Rangsit University, Pathum Thani 12000, Thailand; ^2^Department of Protozoology, Faculty of Tropical Medicine, Mahidol University, Bangkok 10400, Thailand; ^3^Division of Microbiology, Department of Central Laboratory and Blood Bank, Faculty of Medicine, Vajira Hospital, Navamindradhiraj University, Bangkok 10300, Thailand

## Abstract

*Citrus reticulata* Blanco and *Citrus aurantifolia* are the edible plants which contain several biological properties including antibacterial activity. The aims of the present study were to determine the chemical compositions and evaluate antibacterial activities of citrus essential oils extracted from the fruit peels of *C. reticulata* (CREO) and *C. aurantifolia* (CAEO), alone and in combination with gentamicin, against a panel of clinically isolated methicillin-resistant *S. aureus* (MRSA) (*n* = 40) and methicillin-susceptible *S. aureus* (MSSA) (*n* = 45). Gas chromatography-mass spectrometry analysis revealed that 12 and 25 compounds were identified in CREO and CAEO with the most predominant compound of limonene (62.9–72.5%). The antibacterial activities were determined by agar disk diffusion and resazurin-based microdilution methods. The results found that almost all MRSA isolates were resistant to ciprofloxacin, erythromycin, and clindamycin, and some isolates were resistant to gentamicin. CREO and CAEO exhibited inhibitory effects toward clinical isolates (MIC: 1.0–32.0 and 8.0–32.0 mg/mL, respectively), with a similar trend to limonene (MIC: 1.0–32.0 mg/mL). However, the higher antibacterial effects were found in CREO and limonene when compared to CAEO (*p* < 0.01). In combination effect, the results showed the synergistic interaction of gentamicin with CREO and limonene on the MRSA and MSSA isolates (FIC indexes: 0.012–0.258 and 0.012–0.375), but that interaction of gentamicin with CAEO was observed only on MRSA (FIC index: 0.012–0.016). These findings demonstrated the potential of these citrus essential oils as natural antibacterial agents that may contribute to reduce the emerging of antimicrobial-resistant bacteria.

## 1. Introduction

*Staphylococcus aureus*, a Gram-positive commensal bacterium, is mainly colonized in the nasal carriage as well as in the skin, axillae, perineum, and pharynx [1]. It can cause a wide variety of infectious diseases, ranging from mild skin and soft tissue infections to life-threatening such as endocarditis, osteomyelitis, pneumonia, and bacteremia. Due to overuse of antibiotics, the emergence of the antibiotic-resistant phenotype has been reported worldwide. Methicillin-resistant *S. aureus* (MRSA), caused by the acquisition of staphylococcal cassette chromosome *mec* (SCC*mec*) which carries the *mecA* gene that is responsible for the production of an altered penicillin-binding protein 2a (PBP2a), thus associated with decreased affinity for all *β*-lactam antibiotics. The relative high burden of hospital-acquired MRSA (HA-MRSA) and community-acquired MRSA (CA-MRSA) is a major concern worldwide. HA-MRSA has a higher mortality rate, an increased duration of hospitalization, and a higher healthcare cost [2]. Vancomycin has been the drug of choice and is currently noticed as the last resort for the treatment of severe MRSA infection [2, 3]. However, nephrotoxicity, hypotension, and hypersensitivity reactions are commonly presented; thus, drug monitoring is required [4]. In this context, the searching for a new candidate of alternative anti-MRSA agent with a lesser toxicity is required, and one of the possible ways to support this goal is the use of plant-derived agents.

The herbal medicinal products have been considered as a natural source for alternative treatment for bacterial infections. *Citrus reticulata* Blanco (commonly known as mandarin orange and tangerine orange) and *Citrus aurantifolia* (commonly known as key lime, common lime, and lime) are edible fruits belonging to the family Rutaceae. They are cultivated in tropical and subtropical regions worldwide. Generally, they can be applied as food and drink flavoring ingredients as they have signature citrus flavoring and scents. *C. aurantifolia* is traditionally used to promote the digestion process and for antidiabetic and antihypercholesterolemic purposes. The oil extracted from the *C. aurantifolia* fruits can be used for relieving cold, asthma, and arthritis [5]. The productivities of *C. reticulata* and *C. aurantifolia* were annually reported at 153,375 and 213,716 tons in Thailand [6]. Several biological properties of crude extracts and essential oils from *Citrus* spp., *C. reticulata* and *C. aurantifolia*, have been reported, including antioxidant [7–11], antibiofilm [12], antibacterial, and antifungal activities [10, 13]. The dried peel and pulp extracts of *C. aurantifolia* from Indonesia possessed antioxidant, antidiabetic, and antibacterial activities against *S. aureus* and *K. pneumoniae* [9].

Additionally, antibacterial activities of crude extracts and essential oils (EOs) from different parts (leaves, stems, roots, flowers, and peels) of *C. reticulata* and *C. aurantifolia* have demonstrated broad-spectrum antibacterial activities toward clinically important pathogens with a major activity against Gram-positive bacteria, especially *S. aureus* [7, 9–11, 14–20]. Previously, some studies have investigated the antistaphylococcal effect of these citrus-derived products, mostly against the reference strains of MRSA and methicillin-susceptible *S. aureus* (MSSA). Bektaš et al. reported the inhibitory effect of orange juices on MSSA ATCC 25923, MRSA NCTC 1249, and clinical isolates of MRSA [21]. Vong et al. reported the inhibitory effect of the fruit juice extract of *C. aurantifolia* from Malaysia against MRSA ATCC 33591 [22]. Chao et al. reported the inhibitory effects of several commercial citrus EOs including *C. aurantifolia* EO which inhibited the growth of MRSA ATCC 700699 [23]. Up to now, there are limited data on anti-MRSA effects of the citrus extracts and EOs toward the clinical isolates, in which more variation in the antimicrobial susceptibility pattern is observed. Therefore, this study aimed to determine the antibacterial activities of the citrus EOs and their major compounds against clinically isolated MRSA and MSSA. In addition, the synergistic effects of citrus EOs and their major compounds to improve the efficacy of the antibacterial agent, gentamicin, against clinically isolated MRSA and MSSA were also investigated.

## 2. Materials and Methods

### 2.1. Plant Materials

The fresh fruits of *C. reticulata* Blanco (mandarin orange) and *C. aurantifolia* (Christm.) Swingle (lime) were collected from Chiang Rai Province, the northernmost of Thailand, in December 2019. The plant samples were identified, and voucher specimens (BCU no. 015859 and BCU no. 015858) were housed at the herbarium of the Department of Botany, Faculty of Science, Chulalongkorn University, Thailand. The fruit peels were ground, suspended in distilled water, and then processed through hydrodistillation for 3 h. Essential oils were separated from the aqueous layer using a micropipette. The oils were dried over anhydrous sodium sulfate, filtered, and stored at 4°C [24]. In this study, the percentage yields of extracted *C. reticulata* essential oil (CREO) and *C. aurantifolia* essential oil (CAEO) were 0.48% and 0.30%, respectively. The extracted EOs with a density of 0.8 g/mL were stored at 4°C and protected from light. A stock solution was prepared at the concentration of 400 mg/mL in dimethyl sulfoxide (DMSO) before use.

### 2.2. Bacterial Organisms

The tested bacterial organisms contained 2 American Type Culture Collection (ATCC) bacterial strains including *Staphylococcus aureus* ATCC 43300 (methicillin-resistant *S. aureus*, MRSA) and *S. aureus* ATCC 25923 (methicillin-susceptible *S. aureus*, MSSA) as well as clinically isolated MRSA (*n* = 40) and MSSA (*n* = 45). The clinical isolates were obtained from the Division of Microbiology, Department of Central Laboratory and Blood Bank, Faculty of Medicine, Vajira Hospital, Navamindradhiraj University, Thailand, and identified by matrix-assisted laser desorption/ionization time-of-flight mass spectrometry (MALDI-TOF MS). These clinical isolates were originally collected from skin, bloodstream, respiratory, and urinary tracts (Table 1). The bacteria were maintained at −70°C and subcultured on blood agar at 37°C overnight prior the assay.

### 2.3. Antibiotic Susceptibility Test

The susceptibilities of 8 antibiotics (Oxoid, England) including cefoxitin (30 *μ*g), ampicillin (10 *μ*g), amoxicillin (30 *μ*g), gentamicin (10 *μ*g), ciprofloxacin (5 *μ*g), erythromycin (5 *μ*g), clindamycin (2 *μ*g), and vancomycin (E-test) were characterized by agar diffusion, according to CLSI (2019). The susceptibility patterns were interpreted by the inhibition zone diameter (IZD). *S. aureus* was considered as MRSA when IZD of cefoxitin ≤21 mm and MSSA when IZD ≥22 mm [25].

### 2.4. GC-MS Analysis

The separation and identification of volatile components of CREO and CAEO were carried out by gas chromatography-mass spectrometry (GC-MS) (GC 7890A/MS 5975C-MSD; Agilent Technologies, CA, USA). The capillary column Mega-5MS (30 m × 0.25 mm × 0.25 *μ*m) was used. The GC conditions were programmed as the injection temperature 250°C, with oven temperature initially set at 50°C for 1 min and then gradually increasing at the rate of 3°C/min up to 250°C and held for 5 min. Helium was used as the carrier gas with a constant flow rate of 1.0 mL/min. The volume of injection was 1 *μ*L of ethanol solution in a split mode (1 : 10). The MS transfer line temperature was set at 250°C with the electron ionization (EI) mode at 70 eV ionization potential. The mass-to-charge (m/z) range was from 40 to 650 m/z. Compounds were further identified by matching their mass spectra fragmentation pattern and retention time with standard reference compounds, compared their MS results with the NIST 2011 library, and stored in the GC/MS database for confirmation.

### 2.5. Agar Disk Diffusion

Agar disk diffusion was performed to screen the *in vitro* antibacterial activities of the EOs as previously described [26]. Sterilized disks (6 mm in diameter) impregnated with 10 *μ*L of each EO or pure limonene (Lot no. MKCD9298; Sigma-Aldrich, St. Louis, MO, USA) were placed on the surface of the Mueller–Hinton agar (MHA; Oxoid, England) plate after tested bacteria (0.5 McFarland unit) were inoculated. The disk containing 10 *μ*L of 4% DMSO and commercial gentamicin disk (10 *μ*g) (Oxoid, England) were used as negative and positive controls, respectively. After incubation at 37°C for 18−24 h, the IZD of the EOs was measured and interpreted following the criteria: no activity, IZD = 6 mm; weak activity, 6 mm < IZD ≤ 12 mm; moderate activity, 12 mm < IZD < 20 mm; and strong activity, IZD > 20 mm [27].

### 2.6. Determination of MIC

The minimum inhibitory concentrations (MICs) of the EOs were evaluated by the resazurin-based 96-well plate microdilution method as previously described with some modifications [28]. Fifty microliters of various concentrations of EOs and limonene were prepared by a serial two-fold dilution with cation-adjusted Mueller–Hinton broth (CAMHB) in a sterile 96-well microplate to obtain the final concentrations ranging from 0.1 to 32.0 mg/mL. In addition, gentamicin supplement was included in the experiment with the final concentration of 0.1 to 256.0 *μ*g/mL. Afterward, 50 *μ*L of tested bacteria was added into each well to obtain a final concentration of 5 × 10^5^ CFU/mL. Only EO dissolved in CAMHB (oil control), only bacterial suspension in CAMHB (bacterial control), and 4% DMSO with bacterial suspension (diluent control) were also included. After incubation at 37°C for 24 h, 5 *μ*L of 0.015% resazurin (Sigma-Aldrich, St. Louis, MO, USA) was added into each well and further incubated for 2 h in the dark. The bacterial growth was visually observed by the change of resazurin natural color (blue-purple) into the reduced form (red-colorless). The MIC was defined by the lowest concentration that completely inhibits the growth of bacteria (no color change). Consequently, one loop of the MIC suspension that showed no color change was cultivated on the MHA plate and further incubated at 37°C for 18−24 h. The minimum bactericidal concentration (MBC) was defined by the lowest concentration that completely kills bacteria on the agar plate. The MIC index (MBC/MIC ratio) was calculated to classify the type of antimicrobial substances and interpreted using the following criteria: bactericidal, MIC index ≤ 4; bacteriostatic, MIC index > 4; and resistance, MIC index ≥ 32 [29].

### 2.7. Checkerboard Titration Assay

The checkerboard titration assay was performed to evaluate the synergistic interaction among EOs and limonene combined with gentamicin against 7 clinical isolates. This method was based on the broth microdilution assay with the final volume of 100 *μ*L. In brief, 25 *μ*L of various concentrations of EOs or limonene ranging from 0.3 to 32.0 mg/mL was prepared by serial 2-fold dilution in the 96-well microplate. In the meanwhile, various concentrations of gentamicin were prepared ranging from 0.001 to 128.0 *μ*g/mL. Then, 25 *μ*L of each concentration of gentamicin was added into each concentration of EO or limonene to perform checkerboard testing. Fifty microliters of tested bacteria were added into each well to obtain a final concentration of 5 × 10^5^ CFU/mL and incubated at 37°C for 18−24 h. Afterward, 5 *μ*L of 0.015% resazurin was added and further incubated for 2 h in the dark. The bacterial growth was visually observed by the color change of resazurin. The combination effect of either EOs or limonene with gentamicin was determined by using the fractional inhibitory concentration index (FICI) value using the following formula:

(1) $$\begin{array}{c} \text{FIC}=\frac{\text{MIC of the EOs},l\text{imonene or gentamicin in combination}}{\text{MIC of the EOs},\text{limonene or gentamicin alone}} \end{array}$$

(2) $$\begin{array}{c} \text{FICI}=\text{FIC of the EOs or limonene}+\text{FIC of gentamicin} \end{array}$$

The interaction was interpreted by using the following criteria: FICI ≤ 0.5, synergy; 0.5 < FICI ≤ 1, additive; 1 < FICI ≤ 4, indifference; and FICI > 4, antagonism [30].

### 2.8. Statistical Analysis

All experiments were performed in triplicate. The data were analyzed with the descriptive statistics, Kruskal–Wallis test, and Mann–Whitney *U* test using the IBM Statistical Package for Social Services (SPSS) version 21.0 (IBM, Armonk, NY, USA). A *p* value <0.05 was considered statistically significant.

## 3. Results

### 3.1. Chemical Composition of EOs

**Figure 1:**
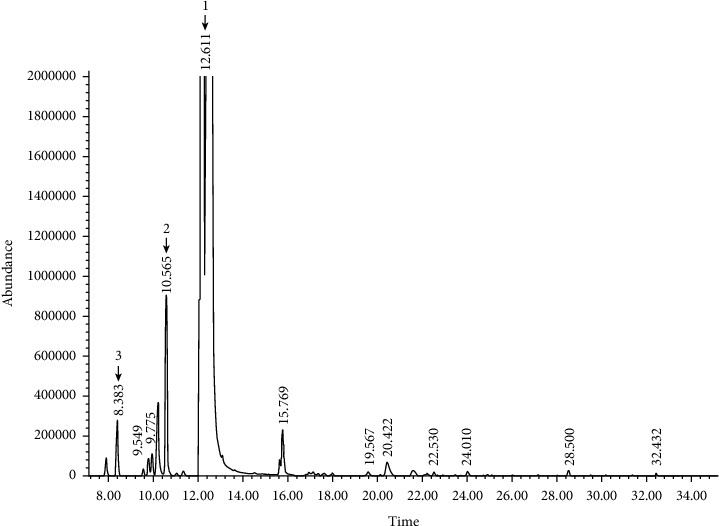
Representative GC-MS chromatogram of the *Citrus reticulata* Blanco essential oil. Major compound peaks were marked as follows: D-limonene (1), *β*-myrcene (2), and *α*-pinene (3).

**Figure 2:**
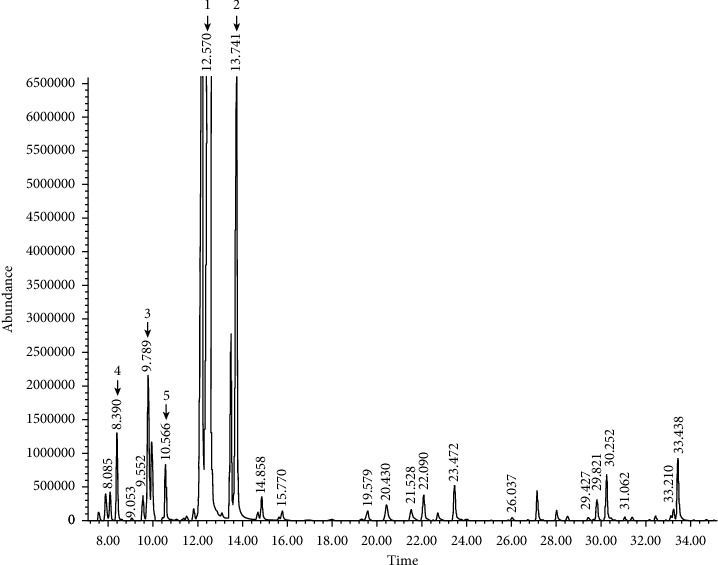
Representative GC-MS chromatogram of the *Citrus aurantifolia* essential oil. Major compound peaks were marked as follows: D-limonene (1), *γ*-terpinene (2), *β*-pinene (3), *α*-pinene (4), and *β*-myrcene (5).

The wide variety of chemical compositions of the citrus EOs, *Citrus reticulata* (CREO) and *Citrus aurantifolia* (CAEO), are presented in Tables 2 and 3, and the GC-MS chromatograms are shown in Figures 1 and 2. Twelve and 25 compounds accounted for 74.8% of the total CREO and 96.8% of the total CAEO, respectively, with predominant monoterpene hydrocarbons (74.2% and 91.6%), followed by oxygenated monoterpenes (0.6% and 2.4%) and sesquiterpene hydrocarbons (0.1% and 2.8%).

In CREO, D-limonene (72.53%) was the major component of monoterpene hydrocarbons followed by *β*-myrcene (1.03%) and *α*-pinene (0.45%), while *β*-linalool (0.34%) and *α*-terpineol (0.18%) were the major components of oxygenated monoterpenes. The compositions of remaining 7 compounds in CREO ranged from 0.01 to 0.12% (Table 2). In CAEO, D-limonene (62.95%) was the major component of monoterpene hydrocarbons followed by *γ*-terpinene (13.30%), *β*-pinene (11.40%), *α*-pinene (1.55%), *β*-myrcene (0.92%), and *α*-thujene/*β*-thujene (0.45%), while *α*-citral (0.74%), *β*-citral (0.55%), and *α*-terpineol (0.45%) were the major components of oxygenated monoterpenes; and *β*-bisabolene (1.13%), *α*-trans-bergamotene (0.80%), and caryophyllene (0.39%) were the major components of sesquiterpene hydrocarbons. The compositions of remaining 13 compounds in CAEO ranged from 0.04 to 0.43% (Table 3).

### 3.2. Antibiotic Susceptibility Pattern

The susceptibility patterns of tested bacteria in this study are shown in Table 4. The susceptibility to cefoxitin (10 *μ*g) was used to classify between MRSA and MSSA. The results showed that *S. aureus* ATCC 43300 and 40 clinical isolates of *S. aureus* were resistant to cefoxitin (IZD: 14.0 ± 1.7 mm and 7.2 ± 2.4 mm, respectively), indicating MRSA phenotype, while *S. aureus* ATCC 25923 and the other 45 clinical isolates were susceptible to cefoxitin (IZD: 28.7 ± 5.0 mm and 30.0 ± 2.3 mm, respectively), indicating MSSA phenotype.

It also showed that MSSA ATCC 25923 was susceptible to all other tested antibiotics including gentamicin (IZD: 26.7 ± 1.2 mm), ciprofloxacin (IZD: 25.0 ± 1.0 mm), erythromycin (IZD: 28.0 ± 1.0 mm), clindamycin (IZD: 30.0 ± 2.1 mm), and vancomycin (MIC: 1.9 ± 0.1 *μ*g/mL), while MRSA ATCC 43300 was susceptible to gentamicin (IZD: 23.0 ± 1.0 mm), ciprofloxacin (IZD: 25.0 ± 3.6 mm), and vancomycin (MIC: 1.0 ± 0.0 *μ*g/mL) but resistant to erythromycin (IZD: 6.0 ± 0.0 mm) and clindamycin (IZD: 6.0 ± 0.0 mm). The IZDs of ampicillin and amoxicillin were compared since there were no interpreting criteria for the susceptibility pattern of these antibiotics, and it showed that MRSA ATCC 43300 was lesser susceptible to ampicillin and amoxicillin than MSSA ATCC 25923 (IZD: 12.0 ± 3.5 mm vs. 33.0 ± 2.6 mm for ampicillin and 14.7 ± 2.3 mm vs. 30.0 ± 5.3 mm for amoxicillin).

The resistant rate to gentamicin among clinical isolates of MRSA (15/40, 37.5%) was higher than that of MSSA (2/45, 4.4%) with the IZDs of 17.7 ± 8.4 mm and 22.3 ± 3.5 mm, respectively (*p* < 0.01). For other antibiotics, MRSA isolates showed high rates of resistance to ciprofloxacin, erythromycin, and clindamycin (76.9–95.0%) when compared to MSSA isolates (6.7–16.7%) with the IZDs of 8.5 ± 7.2 mm, 9.7 ± 7.9 mm, and 9.7 ± 8.1 mm for MRSA and the IZDs of 25.2 ± 6.3 mm, 23.0 ± 8.6 mm, and 23.3 ± 7.7 mm for MSSA (*p* < 0.01). In this study, all MRSA (40/40, 100%) were susceptible to vancomycin, which is a last-resort antibiotic for multidrug-resistant (MDR) bacteria at MIC 0.5 ± 0.2 *μ*g/mL. As the expected results, MRSA isolates were less susceptible to *β*-lactam antibiotics, ampicillin and amoxicillin, when compared to MSSA isolates with the IZDs of 9.7 ± 5.8 mm vs. 21.9 ± 8.6 mm for ampicillin and 10.4 ± 6.6 mm vs. 25.6 ± 6.9 mm for amoxicillin (*p* < 0.01). All these together, it demonstrated that MRSA isolates seem to be resistant to all tested antibiotics except for vancomycin.

In addition, MDR *S. aureus*, which is classified by the resistant pattern to at least one agent among at least three antibiotic classes, was found at high rate among MRSA isolates (31/40, 77.5%), but only one was observed among MSSA isolates (1/45, 2.2%). This MDR MSSA isolate was resistant to all antibiotics except cefoxitin. Beside the resistance to cefoxitin (31/31, 100%), the most common antibiotic resistance of MDR MRSA was erythromycin (31/31, 100%), followed by ciprofloxacin (30/31, 96.8%), clindamycin (30/31, 96.8%), and gentamicin (15/31, 48.4%) (data not shown).

### 3.3. Antibacterial Activity of Citrus EOs and Limonene

The values of IZD, MIC, and MBC of the citrus EOs and pure limonene against tested bacteria are shown in Table 5. In this study, sterile disk containing 4% DMSO had no inhibitory effect, while commercial gentamicin disk (10 *μ*g) had the effect toward all *S. aureus* isolates including laboratory strains. By agar disk diffusion, the results of antibacterial activity of CREO, CAEO, and limonene revealed that these agents had an inhibitory effect against almost all tested bacteria including MRSA and MSSA. CREO exhibited antibacterial activity against both MRSA ATCC 43300 and MSSA ATCC 25923 with the IZDs of 11.3 ± 1.5 mm and 11.7 ± 1.5 mm, respectively. Limonene exhibited antibacterial activity against both MRSA ATCC 43300 and MSSA ATCC 25923 with the IZDs of 12.3 ± 1.5 mm and 13.0 ± 1.7 mm, respectively. On the contrary, CAEO exhibited antibacterial activity against MSSA ATCC 25923 (IZD: 10.3 ± 3.8 mm) but not MRSA ATCC 43300 (IZD: 6.0 ± 0.0 mm).

For clinical isolates, the IZDs of CREO against MRSA and MSSA were 11.7 ± 3.1 mm and 12.0 ± 2.5 mm, respectively. The IZDs of CAEO were 11.6 ± 3.9 mm and 10.0 ± 2.9 mm for MRSA and MSSA, while those of limonene were 14.5 ± 3.9 mm and 14.4 ± 2.9 mm for MRSA and MSSA, respectively. However, 2 clinical isolates, 1 MRSA and 1 MSSA, had no activity (IZD: 6.0 ± 0.0 mm) against CREO, whereas 5 clinical isolates, 3 MRSA and 2 MSSA, had no activity against CAEO. All clinical isolates were inhibited by limonene. By the results of agar disk diffusion, it also demonstrated that CREO inhibited clinically isolated MSSA better than CAEO with a significant difference (*p* < 0.01). However, the significant difference was not observed in MRSA isolates (*p* > 0.05). For limonene, the major compound of CREO and CAEO, the IZDs against MRSA and MSSA were significantly higher than those of CREO and CAEO (both *p* < 0.01). The results indicated that limonene was the highest effective agent following CREO and CAEO.

When the levels of IZD were interpreted according to a previous study [27], it demonstrated various degrees of antibacterial activities of CREO, CAEO, and limonene observed among clinical isolates of *S. aureus*. CREO and CAEO exhibited weak activities against the clinical isolates of MRSA (57.5% and 47.5%, respectively), followed by moderate activity (40.0% and 42.5%, respectively). Likewise, CREO and CAEO exhibited weak activities against the clinical MSSA isolates (55.5% and 68.8%, respectively), followed by moderate activity (42.3% and 26.7%, respectively). On the contrary, limonene exhibited moderate activities (55.0% and 68.9%), followed by weak activity (35.0% and 26.7%), against the clinical MRSA and MSSA isolates, respectively. As the expected result, it showed that pure limonene, which is considered as the major composition of CREO and CAEO, efficiently inhibited all tested bacteria including MRSA and MSSA, with a moderate activity.

According to the microdilution method, oil control, bacterial control, and diluent control showed that no contamination in the tested oils, growth ability of tested bacteria, and no inhibitory effect occurred by the diluent, respectively. In this study, the results found that MRSA ATCC 43300 and MSSA ATCC 25923 were inhibited by CREO with MIC equal to 4.0 ± 0.0 mg/mL. For clinical isolates, CREO inhibited MRSA (MIC: 13.6 ± 10.8 mg/mL) and MSSA (MIC: 12.6 ± 8.5 mg/mL) with no significant difference (*p* > 0.05). For another EO, the MIC values of CAEO demonstrated antibacterial activities against 2 ATCC strains lower than those of CREO. It inhibited MRSA ATCC 43300 and MSSA ATCC 25923 with MIC equal to 8.0 ± 0.0 mg/mL. In the same manner as CREO, CAEO also inhibited clinically isolated MRSA and MSSA (MIC: 21.6 ± 8.6 mg/mL and 20.6 ± 8.2 mg/mL, respectively) with no significant difference (*p* > 0.05). However, the minimum concentration of CREO which inhibited bacterial growth was lower than that of CAEO with a significant difference (*p* < 0.01). It demonstrated that CREO inhibited clinically isolated *S. aureus* better than CAEO. For limonene, the MIC values demonstrated that it inhibited MRSA ATCC 43300 and MSSA ATCC 25923 ranging from 2.0 to 4.0 mg/mL. It also inhibited clinically isolated MRSA (MIC: 7.9 ± 5.1 mg/mL) and MSSA (MIC: 9.5 ± 8.4 mg/mL) with no significant difference (*p* > 0.05). In addition, the MIC values of limonene against MRSA and MSSA isolates were significantly different when compared to those of CAEO (*p* < 0.01). Like the results of agar disk diffusion, the results of MIC indicated that antibacterial activities toward all tested bacteria were sorted in the descending order as follows: limonene, CREO, and CAEO. Regarding MIC indexes, the citrus EOs and limonene tend to be acting as bactericidal agents towards MRSA and MSSA isolates (MIC index: 1.3–2.0).

### 3.4. Synergistic Activities of Gentamicin Combined with Citrus EOs and Limonene

The combination interaction of gentamicin, which is a standard antibiotic against *S. aureus*, combined with citrus EOs or limonene is presented in Table 6. In this study, the synergistic effect was evaluated against 7 clinical isolates containing 5 isolates of MRSA and 2 isolates of MSSA using the checkerboard titration assay. The results demonstrated that gentamicin in combination with CREO showed synergistic interaction (FICI: 0.012–0.258) among the most MRSA (3/5, 60.0%) and MSSA isolates (1/2, 50.0%). On the contrary, gentamicin in combination with CAEO showed synergistic interaction (FICI: 0.012–0.016) in only 2 isolates of MRSA (2/5, 40.0%) and none in MSSA isolates (0/2, 0.0%). Likewise, gentamicin in combination with limonene showed synergistic interaction (FICI: 0.012–0.375) in almost all MRSA isolates (4/5, 80.0%), but none in MSSA isolates (0/2, 0.0%). However, no antagonistic effect was observed in the combination of gentamicin with CREO, CAEO, or limonene. These findings revealed the synergistic effect of gentamicin with the citrus EOs or limonene on clinical isolates of *S. aureus*, especially MRSA.

## 4. Discussion

The fruit peels of *Citrus* spp. are byproducts remained in manufacturing processes of several products such as orange juice and lime juice. The development of these byproducts should be approved to increase their worth. One advantage of the fruit peels is that some can be developed as natural antibiotics. Antibacterial activity of the *Citrus* spp. on a panel of clinical isolates of the resistant and susceptible strains of *S. aureus* has not been reported. This study was therefore interested to apply EOs extracted from the fruit peels of *C. reticulata* (CREO) and *C. aurantifolia* (CAEO) to fight against the clinical strains of resistant and susceptible *S. aureus*, with our expectation to increase the economic value of their byproducts and reduce the emerging of antibiotic-resistant *S. aureus*. Additionally, this study determined the antibacterial activities of these two citrus essential oils, CREO and CAEO, and their major components in both single effect and combination interaction with gentamicin.

The extraction yields of CREO (0.48%) and CAEO (0.30%) were in similar ranges of amount with the previous studies (0.22–0.57%) [13, 14]. However, this is difficult to make a comparison since there are many variation factors influenced to the yields of EOs such as climate, geographic distribution, genetics of the plant, the part of the plant used, the degree of freshness, the drying period, and the extraction method [31]. The present study showed that CREO and CAEO are rich in monoterpenes with the major component being D-limonene (72.53% in CREO and 62.95% in CAEO). This is similar to the previous study review that mentioned the contents of limonene in CREO and CAEO of 67.0 to 86.0% [7, 11, 14, 20, 32]. However, the contents of limonene found in CREO and CAEO in this study were higher than previous studies that reported 29.3 to 58.9% of limonene [16, 17, 33]. In fact, the chemical compositions of EOs vary depending on origin, genetic background, season, climate, age, ripening stage, and method of extraction [10].

This study used a panel of clinical Gram-positive strains with different susceptibility profiles to evaluate antibacterial activities of citrus EOs. The chosen bacterial strain was *S. aureus*, including MSSA, MRSA, and MDR phenotypes. It showed that 77.5% of MRSA was MDR. In addition, almost all MRSA were less susceptible than MSSA in various antibiotics such as ampicillin (*β*-lactam), amoxicillin (*β*-lactam), ciprofloxacin (fluoroquinolone), erythromycin (macrolide), and clindamycin (lincosamide). Therefore, these antibiotics could not be used for the treatment of MRSA infections. Fortunately, some MRSA were still susceptible to gentamicin (25/40), and vancomycin-resistant isolates were not observed in this study. However, the emergence of vancomycin-resistant *S. aureus* (VRSA) has been reported. The prevalence of VRSA was 1.2% in Asia, 1.1% in Europe, 3.6% in America, and 2.5% in Africa [34]. The prevalence might be increased in the future. To reduce the usage of vancomycin, the combinations of EOs and limonene with gentamicin were evaluated.

In this study, it appeared that CREO, which contained 72.53% of limonene, had higher effectiveness against MRSA and MSSA strains than that observed in CAEO, which contained 62.95% of limonene; in addition, purified limonene (97%) also showed a promising effect. In addition, a higher concentration of limonene in CREO leads to a better synergistic effect than that of CAEO. Based on these observations, it indicated that limonene had an influence on the antibacterial potential of EOs. This is in agreement to previous reports [16, 20, 35–37] but in contrast to some other studies [14, 31, 38]. The antibacterial mechanism of citrus EOs has been described by a previous study. A possible mechanism was cell wall disruption by citrus EOs on MRSA [39]. This study firstly demonstrated the synergistic effects of CREO, CAEO, and limonene with gentamicin on clinical isolates of MRSA and MSSA. This study proposed that the antibacterial activity of the citrus EOs could attribute to their lipophilicity property and the synergy between their major monoterpene hydrocarbons, limonene, and other minor components such as linalool which has been considered to be an antibacterial agent [37]. The mode of action of EOs could be due to the diffusion and accumulation of the oil in the bacterial cell membrane and then increasing cell membrane permeability, leading to cell lysis and leakage of intracellular components. In addition, the disturbance of the cell membrane may disturb vital processes such as energy conversion, nutrient processing, the synthesis of structural macromolecules, and the secretion of growth regulators [16, 20]. In addition, limonene is believed to accumulate in the bacterial cell membrane and cause the loss of membrane integrity, dissipation of the proton motive force, inhibition of respiration, and ion transport processes [7, 37, 40]. In fact, there are several gentamicin-resistant mechanisms. One resistant mechanism is reducing uptake or decreased cell permeability. The proposed mechanism of synergism is bacterial membrane disruption by the EOs, leading to easier diffusion of gentamicin, an aminoglycoside, across the bacterial membrane. Consequently, this aminoglycoside is able to inhibit bacterial protein synthesis by binding to the 30S ribosomal subunit.

Both CREO and CAEO had an inhibitory effect towards MRSA and MSSA isolated from various types of clinical specimens including skin, bloodstream, respiratory, and urinary tracts (data not shown). It implied that these EOs can be applied as antibacterial agents in several products, such as handwashes and nasal or oral sprays, as well as in several routes of exposure via topical, inhalation, or oral routes. The citrus EOs have been classified as Generally Recognized As Safe (GRAS) [41]. Aumeeruddy-Elalf et al. reported that hydrodistilled essential oils from *Citrus* spp. fruit peels (*C. reticulata*) have no cytotoxicity to human cells [12]. Lime and mandarin essential oils up to 100% had no irritating and sensitizing effects on humans. Acute dermal LD_50_ of lime EO in rabbits and that oral LD_50_ in rats were equal to >5 g/kg [10]. By this strategy, it can minimize the adverse effects of gentamicin in view of reducing the treatment dosage to the resistant bacteria, including reducing treatment costs and providing a therapeutic option with greater antimicrobial potential. Further studies on the precise mode of action, therapeutic dosage, tolerability, and safety of the EOs are necessary to provide therapeutic usage of EOs and in combination with antibiotics.

## 5. Conclusions

This study demonstrated that EOs extracted from *C. reticulata* and *C. aurantifolia* exhibited antibacterial activities against clinical isolates of *S. aureus*, both MRSA and MSSA. The synergistic effects of EOs with gentamicin toward the clinical isolates of MRSA were also revealed. The usage of these EOs would directly inhibit both susceptible and resistant bacteria and indirectly delay the emergence of bacterial resistance, hence the potential of plant-derived antibacterial agents to be used as a complementary therapy with the established antibiotics that would allow for dose reduction of the antibiotic, thereby delaying and reducing the emergence of antibiotic-resistant strains as well as minimizing the possible side effects.

## Figures and Tables

**Table 1:** Clinical sources of tested bacteria used in this study.

Sources	No. (%)	
	MRSA	MSSA
Pus/wound	9 (22.5)	16 (35.5)
Hemoculture	8 (20.0)	13 (28.9)
Sputum	17 (42.5)	13 (28.9)
Urine/stool	6 (15.0)	3 (6.7)

Total	40 (100)	45 (100)

**Table 2:** Chemical compositions of the *Citrus reticulata* Blanco essential oil.

No.	Compounds	Retention time (min)	Retention index^*∗*^	Composition (%)	Quality
1	*α*-Pinene	8.383	936	0.45	97
2	Sabinene	9.549	966	0.04	94
3	*β*-Pinene	9.776	972	0.12	97
4	*β*-Myrcene	10.562	992	1.03	91
5	**D-Limonene**	**12.575**	**1039**	**72.53**	**99**
6	*β*-Linalool	15.768	1111	0.34	83
7	Terpinen-4-ol	19.567	1193	0.04	94
8	*α*-Terpineol	20.422	1212	0.18	90
9	Carvone	22.532	1258	0.03	94
10	Perillal	24.015	1291	0.02	90
11	(-)-*β*-Elemene	28.504	1394	0.04	90
12	D-Germacrene	32.434	1488	0.01	96

^*∗*^Retention index relative to *n*-alkanes (C8-C40) on the Mega-5MS column. The bold values indicate the representative data of the major compound.

**Table 3:** Chemical compositions of the *Citrus aurantifolia* essential oil.

No.	Compounds	Retention time (min)	Retention index^*∗*^	Composition (%)	Quality
1	Thujene	8.087	928	0.45	91
2	*α*-Pinene	8.390	936	1.55	98
3	Camphene	9.059	953	0.04	96
4	Sabinene	9.548	966	0.29	91
5	***β*** **-Pinene**	**9.790**	**972**	**11.41**	**97**
6	*β*-Myrcene	10.569	992	0.92	91
7	*α*-Phellandrene	11.369	1012	0.04	91
8	*α*-Terpinene	11.824	1022	0.23	96
9	**D-Limonene**	**12.575**	**1039**	**62.95**	**98**
10	***γ*** **-Terpinene**	**13.741**	**1065**	**13.30**	**96**
11	Terpinolene	14.858	1091	0.43	97
12	*β*-Linalool	15.829	1111	0.19	80
13	Terpinen-4-ol	19.581	1194	0.24	93
14	*α*-Terpineol	20.422	1212	0.45	72
15	cis-Geraniol	21.532	1236	0.26	93
16	*β*-Citral	22.091	1249	0.55	87
17	*α*-Citral	23.47	1279	0.74	97
18	*δ*-Elemene	26.042	1337	0.06	98
19	*α*-Bergamotene	29.435	1416	0.07	98
20	Caryophyllene	29.821	1425	0.39	99
21	*α*-trans-Bergamotene	30.255	1436	0.80	91
22	Terpinolene	14.858	1091	0.43	97
23	*β*-Linalool	15.829	1111	0.19	80
24	Terpinen-4-ol	19.581	1194	0.24	93
25	*α*-Terpineol	20.422	1212	0.45	72

^*∗*^Retention index relative to *n*-alkanes (C8-C40) on the Mega-5MS column. The bold values indicate the representative data of the major compound.

**Table 4:** Antibiotic susceptibility pattern of tested bacteria.

Bacterial strains	IZD (mm)							MIC^*∗*^ (*μ*g/mL)
	FOX	CN	CIP	ERY	DA	AMP	AMC	VA
Reference strains								
*S. aureus* ATCC 43300	14.0 ± 1.7 (R)	23.0 ± 1.0 (S)	25.0 ± 3.6 (S)	6.0 ± 0.0 (R)	6.0 ± 0.0 (R)	12.0 ± 3.5	14.7 ± 2.3	1.0 ± 0.0 (S)
*S. aureus* ATCC 25923	28.7 ± 5.0 (S)	26.7 ± 1.2 (S)	25.0 ± 1.0 (S)	28.0 ± 1.0 (S)	30.3 ± 2.1 (S)	33.0 ± 2.6	30.0 ± 5.3	1.9 ± 0.1 (S)

MRSA isolates (*n* = 40)	7.2 ± 2.4^*∗∗*^	17.7 ± 8.4^*∗∗*^	8.5 ± 7.2^*∗∗*^	9.7 ± 7.9^*∗∗*^	9.7 ± 8.1^*∗∗*^	9.7 ± 5.8^*∗∗*^	10.4 ± 6.6^*∗∗*^	0.5 ± 0.2
No. of resistance (%)	40 (100%)	15 (37.5%)	38 (95.0%)	31 (77.5%)	30 (76.9%)	NA	NA	0 (0%)
No. of intermediate (%)	0 (0%)	0 (0%)	0 (0%)	0 (0%)	0 (0%)	NA	NA	0 (0%)
No. of sensitive (%)	0 (0%)	25 (62.5%)	2 (5%)	9 (22.5%)	9 (23.1%)	NA	NA	40 (100%)

MSSA isolates (*n* = 45)	30.0 ± 2.3	22.3 ± 3.5	25.2 ± 6.3	23.0 ± 8.6	23.3 ± 7.7	21.9 ± 8.6	25.6 ± 6.9	NA
No. of resistance (%)	0 (0%)	2 (4.4%)	3 (6.7%)	4 (16.7%)	4 (14.8%)	NA	NA	NA
No. of intermediate (%)	0 (0%)	0 (0%)	3 (6.7%)	3 (12.5%)	2 (7.4%)	NA	NA	NA
No. of sensitive (%)	45 (100%)	43 (95.6%)	39 (86.6%)	17 (70.8%)	21 (77.8%)	NA	NA	NA

Values are expressed as mean ± SD of triplicate experiments. AMP: ampicillin (10 *μ*g); AMC: amoxicillin (30 *μ*g); FOX: cefoxitin (30 *μ*g); CN: gentamicin (10 *μ*g); CIP: ciprofloxacin (5 *μ*g); ERY: erythromycin (15 *μ*g); DA: clindamycin (2 *μ*g); VA: vancomycin; S: susceptible; R: resistant; NA: not applicable. Susceptibilities of *S. aureus* against AMP and AMC were not interpreted by CLSI (2019). ^∗^Vancomycin-susceptible S. aureus (VSSA), MIC ≤2 μg/mL. ^∗∗^*p* < 0.01 (Mann–Whitney U test), statistically significant when compared to the MSSA isolate.

**Table 5:** Antibacterial activities of the citrus essential oils and limonene against MRSA and MSSA by agar disk diffusion and microdilution methods.

Bacterial strains	CREO				CAEO				Limonene			
	IZD (mm)	MIC (mg/mL)	MBC (mg/mL)	MIC index	IZD (mm)	MIC (mg/mL)	MBC (mg/mL)	MIC index	IZD (mm)	MIC (mg/mL)	MBC (mg/mL)	MIC index
Reference strains												
*S. aureus* ATCC 43300	11.3 ± 1.5 (W)	4.0 ± 0.0	4.0 ± 0.0	1.0	6.0 ± 0.0 (N)	8.0 ± 0.0	12.0 ± 5.7	1.5	12.3 ± 1.5 (M)	3.0 ± 0.4	8.0 ± 0.0	3.0
*S. aureus* ATCC 25923	11.7 ± 1.5 (W)	4.0 ± 0.0	4.0 ± 0.0	1.0	10.3 ± 3.8 (W)	8.0 ± 0.0	12.0 ± 5.7	1.5	13.0 ± 1.7 (M)	3.0 ± 1.4	3.0 ± 1.4	1.0
MRSA isolates (*n* = 40)	11.7 ± 3.1^*∗*^	13.6 ± 10.8^*∗*,*∗∗*^	19.4 ± 11.3	2.0 ± 1.4	11.6 ± 3.9^*∗*^	21.6 ± 8.6^*∗*^	27.4 ± 17.3	1.5 ± 0.5	14.5 ± 3.9	7.9 ± 5.1	12.9 ± 8.0	1.9 ± 0.9
No activity	1 (2.5%)				3 (7.5%)				0 (0%)			
Weak activity	23 (57.5%)				19 (47.5%)				14 (35.0%)			
Moderate activity	16 (40.0%)				17 (42.5%)				22 (55.0%)			
Strong activity	0 (0%)				1 (2.5%)				4 (10.0%)			
MSSA isolates (*n* = 45)	12.0 ± 2.5^*∗*,*∗∗*^	12.6 ± 8.5^*∗*,*∗∗*^	17.7 ± 10.5	1.6 ± 0.8	10.9 ± 2.9^*∗*^	20.6 ± 8.2^*∗*^	23.6 ± 9.7	1.3 ± 0.6	14.4 ± 2.9	9.5 ± 8.4	13.3 ± 8.9	1.9 ± 1.2
No activity	1 (2.2%)				2 (4.5%)				0 (0%)			
Weak activity	25 (55.5%)				31 (68.8%)				12 (26.7%)			
Moderate activity	19 (42.3%)				12 (26.7%)				31 (68.9%)			
Strong activity	0 (0%)				0 (0%)				2 (4.4%)			

Values are expressed as mean ± SD of triplicate experiments. N: no activity; W: weak activity; M: moderate activity; S: strong activity. ^∗^*p* < 0.01 (Mann–Whitney *U* test), statistically significant when compared to limonene. ^∗∗^*p* < 0.01 (Mann–Whitney *U* test), statistically significant when compared to CAEO.

**Table 6:** Combination interaction of the citrus EOs and limonene with gentamicin on MRSA and MSSA by the checkerboard titration method.

Isolate no.	Type	Compounds	MIC^*∗*^		FIC^*∗*^	FIC index	Interaction
			Alone	Combination			
S321	MRSA	CREO	32.0 ± 0.0	0.25	0.008	0.012	Synergy
		Gentamicin	0.5 ± 0.0	0.002	0.004		
		CAEO	32.0 ± 0.0	0.25	0.008	0.012	Synergy
		Gentamicin	0.5 ± 0.0	0.002	0.004		
		Limonene	24.0 ± 11.3	0.25	0.010	0.012	Synergy
		Gentamicin	0.5 ± 0.0	0.001	0.004		

S171	MRSA	CREO	32.0 ± 0.0	0.25	0.008	0.016	Synergy
		Gentamicin	0.25 ± 0.0	0.002	0.008		
		CAEO	32.0 ± 0.0	0.25	0.008	0.016	Synergy
		Gentamicin	0.25 ± 0.0	0.002	0.008		
		Limonene	12.0 ± 5.7	0.25	0.021	0.029	Synergy
		Gentamicin	0.25 ± 0.0	0.002	0.008		

G445	MRSA	CREO	12.0 ± 5.7	4.00	0.333	0.583	Additive
		Gentamicin	64.0 ± 0.0	16.00	0.250		
		CAEO	16.0 ± 0.0	8.00	0.500	1.000	Additive
		Gentamicin	64.0 ± 0.0	32.00	0.250		
		Limonene	8.0 ± 0.0	2.00	0.250	0.375	Synergy
		Gentamicin	64.0 ± 0.0	8.00	0.250		

R569	MRSA	CREO	3.0 ± 1.4	4.00	1.333	1.334	Indifference
		Gentamicin	256.0 ± 0.0	0.125	0.001		
		CAEO	8.0 ± 0.0	8.00	1.000	1.001	Additive
		Gentamicin	256.0 ± 0.0	0.25	0.001		
		Limonene	2.0 ± 0.0	0.50	0.250	0.250	Synergy
		Gentamicin	256.0 ± 0.0	0.016	0.0005		

R201	MRSA	CREO	32.0 ± 0.0	8.00	0.250	0.258	Synergy
		Gentamicin	128.0 ± 0.0	1.00	0.008		
		CAEO	16.0 ± 0.0	16.00	1.000	1.004	Additive
		Gentamicin	128.0 ± 0.0	0.50	0.008		
		Limonene	6.0 ± 2.8	4.00	0.667	0.668	Additive
		Gentamicin	128.0 ± 0.0	0.125	0.008		

R403	MSSA	CREO	6.0 ± 2.8	4.00	0.667	0.671	Additive
		Gentamicin	256.0 ± 0.0	1.00	0.004		
		CAEO	8.0 ± 0.0	8.00	1.000	1.004	Additive
		Gentamicin	256.0 ± 0.0	1.00	0.004		
		Limonene	15.0 ± 0.7	2.00	1.333	1.334	Indifference
		Gentamicin	256.0 ± 0.0	0.063	0.004		

H1025	MSSA	CREO	24.0 ± 11.3	4.00	0.167	0.168	Synergy
		Gentamicin	48.0 ± 22.6	0.063	0.001		
		CAEO	16.0 ± 0.0	16.00	1.000	1.167	Indifference
		Gentamicin	48.0 ± 22.6	8.00	0.001		
		Limonene	6.0 ± 2.8	4.00	0.667	0.688	Additive
		Gentamicin	48.0 ± 22.6	1.00	0.001		

Values are expressed as mean ± SD of duplicate experiments. Clinical sources are as follows: S: pus/wound; H: hemoculture; R: sputum; G: urine/stool. ^∗^MIC and FIC of CREO, CAEO, and limonene are expressed in mg/mL, whereas those of gentamicin are expressed in *μ*g/mL.

## Data Availability

All data are included within the manuscript as tables and figures.
